# HIV viral load suppression before and after COVID-19 in Kinshasa and Haut Katanga, Democratic Republic of the Congo

**DOI:** 10.4102/sajhivmed.v23i1.1421

**Published:** 2022-10-28

**Authors:** Gulzar H. Shah, Gina D. Etheredge, Stacy W. Smallwood, Lievain Maluantesa, Kristie C. Waterfield, Osaremhen Ikhile, John Ditekemena, Elodie Engetele, Elizabeth Ayangunna, Astrid Mulenga, Bernard Bossiky

**Affiliations:** 1 Department of Health Policy and Community Health, Jiann-Ping Hsu College of Public Health, Georgia Southern University, Statesboro, United States of America; 2 FHI 360, Washington, United States of America; 3 Jiann-Ping Hsu College of Public Health, Georgia Southern University, Statesboro, United States of America; 4 FHI 360, Kinshasa, Democratic Republic of the Congo; 5 National Multisectoral HIV/AIDS program (PNMLS), HIV Program, Presidency of DRC, Democratic Republic of the Congo

**Keywords:** HIV, viral load, antiretroviral treatment, COVID-19, Democratic Republic of Congo, PLHIV

## Abstract

**Background:**

The coronavirus disease 2019 (COVID-19) pandemic resulted in unique programmatic opportunities to test hypotheses related to the initiation of antiretroviral treatment (ART) and viral load (VL) suppression during a global health crisis, which would not otherwise have been possible.

**Objectives:**

To generate practice-relevant evidence on the impact of initiating ART pre-COVID-19 versus during the COVID-19 pandemic on HIV VL.

**Method:**

Logistic regression was performed on data covering 6596 persons with HIV whose VL data were available, out of 36 585 persons who were initiated on ART between 01 April 2019 and 30 March 2021.

**Results:**

After controlling for covariates such as age, gender, duration on ART, tuberculosis status at the time of the last visit, and rural vs urban status, the odds of having a VL < 1000 copies/mL were significantly higher for clients who started ART during the COVID-19 pandemic than the year before COVID-19 (adjusted odds ratio [AOR]: 2.50; confidence interval [CI]: 1.55–4.01; *P* < 0.001). Odds of having a VL < 1000 copies/mL were also significantly higher among female participants than male (AOR: 1.23; CI: 1.02–1.48), among patients attending rural clinics compared to those attending urban clinics (AOR: 1.83; CI: 1.47–2.28), and in clients who were 15 years or older at the time of their last visit (AOR: 1.50; CI: 1.07–2.11).

**Conclusion:**

Viral loads did not deteriorate despite pandemic-induced changes in HIV services such as the expansion of multi-month dispensing (MMD), which may have played a protective role regardless of the general negative impacts of response to the COVID-19 crises on communities and individuals.

**What this study adds:**

This research capitalises on the natural experiment of COVID-19-related changes in HIV services and provides new practice-relevant research evidence.

## Introduction

The coronavirus disease 2019 (COVID-19) pandemic has had significant direct effects on the health and well-being of individuals, families, and societies; it has also had significant indirect effects on health through the disruption of health programmes and systems. The combination of COVID-19’s direct and indirect effects on health has been especially profound for people living with HIV (PLHIV). The importance of PLHIV being on an antiretroviral treatment (ART) has been well established, contributing to viral load (VL) suppression as well as reduced odds of advanced HIV disease and mortality.^1,2^

Viral load suppression below 1000 copies/mL is an important indicator of successful ART treatment. Innovative techniques to reduce the risk of VL non-supression are of importance during COVID-19.^3^ To maintain the progress made thus far in controlling the HIV epidemic, the World Health Organization (WHO) issued recommendations to HIV programmes, services, and clinics to ensure uninterrupted HIV care, such as visit frequency, VL testing, and the dispensing of ART. The WHO recommendations include VL monitoring being regularly conducted after PLHIV are placed on ART, with the first VL test six months after ART initiation and annual testing thereafter.^4^ In March 2020, the WHO reiterated their recommendation of less frequent clinic visits for PLHIV that have stable VLs by coupling these visits with the adoption of multi-month prescription (MMP) including 3-month or 6-month multi-month dispensing (MMD-3/MMD-6).^5,6^ This was to be made possible through decentralised distribution which included community pharmacies, automated dispensing, and private hospital models.^7^ In the Democratic Republic of Congo (DRC), the government took precautions to avoid supply stock-outs.^8,9^

Parallels may be drawn between HIV epidemic control and approaches to maintain VL suppression before the COVID-19 pandemic and during the COVID-19 pandemic using differentiated care models, also known as differentiated service delivery.^10^ Differentiated service delivery is a client-centred approach that simplifies and adapts HIV services across the treatment cascade to reflect the preferences and expectations of various groups of PLHIV while reducing unnecessary burdens on the health system.^10^ By providing differentiated service delivery, the health system can reallocate resources to those most in need. Differentiated service delivery applies across the HIV continuum to all three of the 95-95-95 targets (95% of PLHIV should know their status; 95% who know their status should be on ART; 95% of those on ART should be virologically suppressed).^10^ Sustaining services that provide adequate VL testing to monitor ART response in patients^3,11^ and clearing backlogs to increase the number of persons tested can maintain HIV control during a COVID-19 pandemic.^3,12^

A study in Atlanta, United States, showed increased cancellation of appointments during the onset of COVID-19; however, ART adherence improved during the initial month of COVID-19 response.^13^ Long-term effects on VL suppression were not reported. A study in Wuhan, China showed that severe COVID-19 may put PLHIV at greater risk of VL rebound.^14^

In a recent study of facilities in sub-Saharan countries, including the DRC, the impact of the COVID-19 measures was an initial decline in the number of people tested for HIV (January 2020 – June 2020); however, they found that despite these measures there was an increase in the number of people who initiated ART, received VL testing, and whose VL was suppressed.^15^ A Nigerian study showed that PLHIV initiating ART increased approximately eightfold from 04 May 2019 (pre-COVID-19) to 26 September 2020 (after the onset of COVID-19), demonstrating the impact of locally adapted interventions such as the Centers for Disease Control and Prevention (CDC) ART Surge programme in reducing HIV morbidity.^16^

Studies conducted in Uganda and the DRC show that being on ART for longer than 12 months^17^ and adherence to ART^18^ were associated with VL suppression. Patient characteristics such as older age, being married, disclosure of HIV status, receiving care in an urban health zone or one supported by the United States President’s Emergency Plan for AIDS Relief (PEPFAR) were all positively associated with viral suppression^17^ while male sex and tuberculosis (TB) co-infection raised the risk of virological failure.^19^ Few studies have examined the effects of a global health pandemic on the suppression of VLs in PLHIV or have simultaneously examined the long-term effects of VL levels of PLHIV pre-COVID-19 and after the onset of COVID-19. In an attempt to fill this knowledge gap, this research aimed to generate practice-relevant evidence about the nature of changes in VL suppression status in the year immediately pre-COVID-19 (01 April 2019 to 09 Apri1 2020) and after the onset of COVID-19 (10 April 2020 to 30 March 2021).

## Methods

### Data

#### Design

This study used a quantitative, retrospective cohort design based on the date of ART initiation reported in secondary data collected primarily for HIV programme administration.

#### Study setting and sample

The study setting included 313 HIV/AIDS clinics in the Haut-Katanga and Kinshasa provinces of the DRC. The study clinics were run by the DRC government as well as private and faith-based organisations that are supported by the implementing partners participating in the national HIV/AIDS programme (PNLS), and the CDC through PEPFAR. These clinics were located in the following 29 health zones of the Kinshasa and Haut Katanga provinces: Binza Ozone, Kafubu, Kambove, Kasenga, Kashobwe, Katuba, Kikula, Kilelabalanda, Kilwa, Kimbanseke, Kingabwa, Kinshasa, Kipushi, Kisanga, Kowe, Likasi, Limete, Lingwala, Lukafu, Masina I, Matete, Montngafula1, Mufunga Sample, Mumbunda, Ndjili, Ngaba, Nsele, Pweto, and Tshamilemba.

Data from the 36 585 PLHIV who initiated ART between 01 April 2019 and 30 March 2021, were obtained from the HIV programme partners, SANRU (Sante Rurale), HPP (Humana People to People) Congo, and ICAP Global Health in April 2021. Of these, only 6596 patients for whom VL data were available were included in the analysis. These data were gathered by the implementing partners using the electronic patient management and clinical database system, TIER.Net, to track HIV counselling, testing, and service delivery. For this research, only a limited number of de-identified data elements were made available.

### Variables

#### Dependent variable

The dependent variable, VL, was coded as a dichotomous variable with two categories: (1) VL < 1000 copies/mL of blood, and (2) VL ≥ 1000 copies/mL. For each patient who had VL test results reported, the result of the last test was the information used.

#### Independent variables

The primary independent variable related to the timing of ART initiation is either before or during the COVID-19 pandemic. The variable was coded as ‘Pre-COVID-19’ if the ART initiation started in the year immediately before COVID-19 (01 April 2019 to 09 Apri1 2020) and ‘During-COVID-19’ if the ART was initiated after the onset of the COVID-19 pandemic (10 April 2020 to 30 March 2021). Demographic characteristics included the patient’s gender and age at the time of their last visit (15 years or older vs < 15 years of age), which is the standard PEPFAR disaggregating and reporting norm for age. In the bivariate analysis, an interaction variable of age and gender was included with the following categories: < 15 years male, < 15 years female, 15–49.9 years male, 15–49.9 years female, ≥ 50 years male, and ≥ 50 years female. The health zone was coded as rural or urban based on the governmental categorisation. The clinical variables included the duration of ART in months (continuous variable), and TB status on the last visit (no TB detected vs TB detected based on treatment or prescription). The category ‘no symptoms’ was recoded as 0, and was labelled as ‘No TB’. The response categories ‘Under medication TB here’, ‘symptoms without sputum’, ‘symptoms with sputum’, and ‘under Rx TB elsewhere’ were coded as 1 and labelled as ‘TB present’.

### Analytical methods

Descriptive statistics such as frequency distribution, percentages, and arithmetic means for all independent and dependent variables were used to provide contextual information about the study participants’ clinical and demographic characteristics. To test the unadjusted associations between each of the categorical independent variables and the dichotomous dependent variable, we used Cramer’s V for nominal by ordinal variables and Somers’ D test for ordinal by ordinal variables. To model the binary outcome VL suppression below 1000 copies/mL after controlling for demographic and clinical covariates, we used multivariable logistic regression. The significance of associations was determined based on *P* ≤ 0.05. All analyses for this study were performed using Statistical Package for Social Sciences (SPSS) version 25.0.^20^

### Ethical considerations

Georgia Southern University’s Institutional Review Board (IRB) approved the study (protocol no. Hl9260), exempting it from a full IRB review. Patient consent was waived because the secondary data were completely de-identified.

## Results

Among the 36 585 PLHIV who initiated ART during the study period, 52.5% initiated ART in the pre-COVID-19 period from 01 April 2019 to 09 April 2020 and 47.5% did so during the COVID-19 period of 10 April 2020 to 30 March 2021 (Table 1). The majority of the persons receiving ART from these clinics were female (60.9%), 74.5% received ART from urban clinics, and 92.6% were 15 years of age or older. Tuberculosis was not detected in 98.9% of the sample. The average duration of ART for clients included in the study sample was 9.5 months.

**TABLE 1:** Descriptive demographic and clinical characteristics of clients receiving antiretroviral therapy (*N* = 36 585).

Demographic and clinical characteristics	*n*	Percentage
**Viral load suppression status (*n* = 6596)**		
Not suppressed (1000+ copies/mL)	497	7.5
Suppressed (< 1000 copies/mL)	6099	92.5
**When ART initiation occsurred**		
Pre-COVID-19	19 213	52.5
During COVID-19	17 372	47.5
**Patient gender**		
Male	14 026	39.1
Female	21 833	60.9
**Rural or urban of the health zone**		
Urban	27 255	74.5
Rural	9330	25.5
**Age at the time of last visit**		
Younger than 15 years	2713	7.4
15 years or older	33 872	92.6
**TB status**		
No TB	35 302	98.9
TB present	376	1.1

Note: The viral load tests were not available for all people living with HIV, because of testing guidelines. Duration on ART (months): *N* = 36 585, mean = 9.54, confidence interval = 9.47–9.60.

ART, antiretroviral treatment; TB, tuberculosis; COVID-19, coronavirus disease 2019.

The endpoint VL values were only available in a subset of 6596 patients. Table 2 presents descriptive statistics for demographic and clinical characteristics of clients receiving ART and with VL values available. The VL was < 1000 copies/mL for 6099 (92.5%) (Table 2). In the subset with available VL 6060 (91.9%) were initiated on ART pre-COVID and 536 (8.1%) were initiated during COVID. The majority of this smaller subset were female (61.6%), received ART from urban clinics (65.4%), and were 15 years or older (94%). Almost all of the patients (99.3%) did not have TB. Patients with available VL were on ART for an average duration of 15.41 months.

**TABLE 2:** Descriptive demographic and clinical characteristics of clients receiving antiretroviral therapy and with endpoint viral load values available (*N* = 6596).

Demographic and clinical characteristics	*n*	Percentage
**Viral load suppression status**		
Not suppressed (1000+ copies/mL)	497	7.5
Suppressed (< 1000 copies/mL)	6099	92.5
**When ART initiation occurred**		
Pre-COVID-19	6060	91.9
During COVID-19	536	8.1
**Patient gender**		
Male	2520	38.4
Female	4039	61.6
**Rural or urban of the health zone**		
Urban	4314	65.4
Rural	2282	34.6
**Age at the time of last visit**		
Younger than 15 years	396	6.0
15 years or older	6200	94.0
**TB status**		
No TB	6480	99.3
TB present	46	0.7

Note: The viral load tests were not available for all people living with HIV, because of testing guidelines. Duration on ART in months: *N* = 6596, mean = 15.41, confidence interval = 15.31–15.50.

ART, antiretroviral treatment; TB, tuberculosis; COVID-19, coronavirus disease 2019.

The results of the Cramer’s V test for the nominal by ordinal bivariate associations and Somers’ D test for ordinal by ordinal associations are presented in Table 3. The proportion of clients with VLs < 1000 copies/mL was higher during the COVID-19 period than pre-COVID-19. Viral load suppression (< 1000 copies/mL) was higher among female patients than male clients. A higher proportion of clients at rural clinics than urban clinics had VLs < 1000 copies/mL. Being 15 years or older at the time of the last visit was significantly associated with having a VL < 1000 copies/mL. The interaction of age and gender variables was also significantly associated at *P* < 0.001 with VL suppression. The percentage of clients with this level of VL suppression increased with age, as 91.8% 15–49.9 years male, 92.9% 15–49.9 years female, 92.7% ≥ 50 years male, and 95.3% ≥ 50 years female had VLs < 1000 copies/mL. The percentage of clients with a VL < 1000 copies/mL did not differ significantly by TB status.

**TABLE 3:** Bivariate analysis of demographic and clinical factors associated with viral load suppression (< 1000 copies/mL) among patients receiving antiretroviral treatment (*N* = 6596).

Demographic and clinical characteristics	Viral load suppression status		*P*
	Not suppressed (%)	Suppressed (%)	
When ART initiation occurred*			**0.003**
Pre-COVID-19	7.8	92.2	
During COVID-19	4.3	95.7	
Patient gender*			**0.047**
Male	8.3	91.7	
Female	6.9	93.1	
Rural or urban of the health zone*			**< 0.001**
Urban	8.9	91.1	
Rural	5.0	95.0	
Age at the time of last visit**			**0.004**
Younger than 15 years	12.1	87.9	
15 years or older	7.2	92.8	
Age at the time of last visit by gender**			**0.001**
< 15 years male	11.5	88.5	
< 15 years female	10.2	89.8	
15–49.9 years male	8.2	91.8	
15–49.9 years female	7.1	92.9	
≥ 50 years male	7.3	92.7	
≥ 50 years female	4.7	95.3	
TB status*			0.805
No TB	7.4	92.6	
TB present	6.5	93.5	

Notes: Bold values show significance at *P* < 0.05. For this analysis, only 6596 patients for whom viral load data were available were retained in the analysis.Number of years on ART: odds ration = 1.0, *P* = 0.566.

ART, antiretroviral treatment; TB, tuberculosis; COVID-19, coronavirus disease 2019.

* , *P*-values are based on Cramer’s V (nominal by ordinal).

** , *P*-values are based on Somers’ D test (ordinal by ordinal).

Logistic regression of variables associated with a suppressed VL a shown in Table 4. Covariates in the regression models included demographic characteristics (age and gender), clinical characteristics (duration of ART and TB status at the time of the last visit), and rural or urban status of the health zone.

**TABLE 4:** Odds ratios from logistic regression model of whether patients on antiretroviral treatment had their viral load < 1000 copies/mL, pre-COVID-19 and during COVID-19, after controlling for other covariates, March 2019 to March 2021.

Variable	AOR*	95% CI for AOR		*P*-value
		LL	UL	
**When ART initiation occurred**				
During COVID-19	**2.50**	1.55	4.01	< 0.001
Pre-COVID-19**	-	-	-	-
**Client’s gender**				
Female	**1.23**	1.02	1.48	0.032
Male**	-	-	-	-
**Urban or rural status of health zone**				
Rural	**1.83**	1.47	2.28	< 0.001
Urban**	-	-	-	-
**Age at the time of last visit**				
15 years or older	**1.50**	1.07	2.11	0.020
< 15 years of age**	-	-	-	-
**TB status on last visit**				
No TB detected	0.82	0.25	2.66	0.741
TB detected based on treatment or medication**	-	-	-	-
Duration on ART in months (continuous variable)	1.02	1.00	1.05	0.085

Notes: For this analysis, only 6596 patients for whom viral load data were available were retained in the analysis. The final model contained 6526 observations after accounting for missing values for some of the independent variables in the model. The variable province was dropped from the analysis because of a high correlation with the variable urban or rural status; keeping both caused the issue of multicollinearity.

*N*, number of observations; AOR, adjusted odds ratios; CI, confidence interval; LL, lower limit; UL, upper limit; ART, antiretroviral treatment; TB, tuberculosis; COVID-19, coronavirus disease 2019.

* , Reference category;

** , Bold values indicate a *P*-value < 0.05.

## Discussion

This study used a quantitative, retrospective cohort design to analyse data from a sample of 313 HIV care clinics in two provinces of the DRC: Haut-Katanga and Kinshasa. Among persons with HIV receiving ART during the roughly 2-year study period – from 01 April 2019 to 09 April 2020 to mark pre-COVID-19 and 10 April 2020 to 30 March 2021 to mark during COVID-19 – overall, the VL was < 1000 copies/mL for 92.5%. In a recent study of the HIV clinics in the same two provinces of the DRC, Shah et al. reported that among persons receiving ART from 01 January 2014 to 31 May 2019, the proportion with VL ≥ 1000 copies/mL was 18.8%.^19^ Perhaps three factors are accountable for this difference: (1) the study population in their study had been on ART for a much longer period (25% had been on ART for over 40 months, whereas the average duration on ART for the current study participants was 9.5 months, which ranged from 0 to 23 months); (2) the Shah et al. study covered the pre-COVID period whereas one of the two years covered in the current study occurred during the COVID-19 pandemic.

Our initial expectation was that the VL would have been adversely affected by the COVID-related changes in HIV services and disruption in people’s lives. Contrary to those expectations based on the general synthesis of the literature that social determinants of health may lead to worse outcomes for persons with HIV during COVID-19,^21^ our study showed that the odds of VL suppression were higher during COVID-19 than during the pre-COVID-19 period. Our findings were similar to a study of 11 sub-Saharan countries (including DRC), that while the number of those who were tested for HIV, received a positive diagnosis, and began ART treatment, declined in the early months of 2020 and then increased in the later months, the percentage of patients who were achieving viral suppression steadily increased.^15^ The possible factors that could have affected these findings include: (1) the governmental COVID-19 lockdowns and restrictions, which affected the movement of the population within the country and provinces,^9,12^ thus potentially keeping the patients closer to the HIV clinics; (2) the change in the differentiated care models for ART that allowed for patients who would not have initially met MMP and MMD criteria to have access to these, which allowed patients aged 2–13 years old to have access to MMD-3 and those 14 years and older to have access to MMD-6.^22,23,24^ While the exact reasons for the difference cannot be fully analysed with the existing data, this finding holds promise for the ability of healthcare organisations to achieve favourable HIV-related outcomes in the face of emergencies and other events that could have adverse impacts on health systems.

In addition to examining the impact of ART initiation (pre-COVID-19 vs during COVID-19) on VLs, we also examined differences in VL based on demographic and clinical characteristics. These findings are consistent with prior studies that show female patients are more likely to obtain VL suppression than male patients, which can be attributable to differences in ART adherence, psychosocial factors, and overall treatment behaviours.^25,26^ The findings are also consistent with a recent study that reported higher VL suppression in older adult patients (> 35 years).^27,28^ However, our study was not consistent with recent findings regarding VL suppression in patients in urban and rural settings. While our study found a higher likelihood of VL suppression in rural areas, other studies have shown the opposite with the higher likelihood being in urban settings, mostly due to the quality of care and access to HIV centres, testing, and ART dispensing.^29,30^ The differences in these studies may be due to the changes in how care was managed during the COVID-19 pandemic^9,12^ or the sociodemographic factors of the patients which demonstrate that most in rural areas are older and adhere to ART when compared to their urban conterparts.^31^

This study’s findings should be interpreted within the context of its limitations. Inherent in research that is based on quantitative analysis of secondary data, the data were originally collected for a different purpose, in this case, HIV services, and programme administration. First, several contextual variables were not available in the secondary data, the availability of which could have increased the predictive power of the independent variables in the model.^32,33^ For instance, treatment interruption is influenced by an array of socio-economic and lifestyle factors not available in our data. Other determinants of treatment interruption included transiency and length of residence in a community with a high prevalence of HIV, number of sexual partners, an so on, which were not available. Second, this study sample is restricted to the 313 HIV clinics supported by the CDC through the PEPFAR programme and therefore the other HIV service outlets not funded by this programme were not included. For the same reason, the other geographic areas in the DRC could not be covered in the sample. Finally, during the pandemic, some patients could not have accessed the treatment and this study limitation may have skewed the results. Regardless of the limitations of this study, the natural experiment created by COVID-19 and a large-scale robust data set from HIV clinical services providers in two provinces has made our findings unique and generalisable to HIV services in the DRC.

## Conclusion

Given the devastating impact of COVID-19 on healthcare services globally, the intent of the current study was to document the adverse impacts of COVID-19 on VLs of persons on ART, by analysing the difference in VL suppression during the pre-COVID period of 01 March 2019 to 09 April 2020, and during the COVID-19 period of 10 April 2020 to 30 March 2021. We concluded that VL levels were not affected adversely by the net effect of the COVID-19-related adjustments in HIV services (such as MMD and MMP) and additional factors such as broader changes in communities, and individuals’ response to the COVID-19 crises. The odds of having a suppressed VL below 1000 copies/mL were 2.5 times higher in the 12 months during COVID-19 compared to the 12 months after the onset of COVID-19. Our findings suggest that programmes may assess which of the beneficial service adjustments, such as MMD and MMP, made during COVID-19 should be continued in the post-COVID-19 times. The existence of disparities in VLs by gender, rural vs urban clinics, and age suggest that the service providers may consider accounting for these disparities in their services. An in-depth study is imperative to determine modifiable risk factors for high VLs in male patients, people younger than 15 years, and those receiving ART from urban clinics. For example, a qualitative inquiry with PLHIV to further elucidate factors associated with medication adherence or other VL management-related behaviours may yield significant insights. HIV programmes may explore how the broader social determinants of health can be leveraged and programme policies may be revised (e.g. MMD-3 or MMD-6 for patients with stable VLs who may not be able to attend frequent clinical visits; initiation of ART on the same visit as an HIV-positive test result) to be efficient partners in efforts to end the global HIV epidemic.
